# Timely completion of vaccination and its determinants among children in northwest, Ethiopia: a multilevel analysis

**DOI:** 10.1186/s12889-020-08935-8

**Published:** 2020-06-11

**Authors:** Zeleke Abebaw Mekonnen, Kassahun Alemu Gelaye, Martin C. Were, Binyam Tilahun

**Affiliations:** 1 Department of Health Informatics, Institute of Public Health, University of Gondar, Gondar, Ethiopia; 2 Health System Directorate, Federal Ministry of Health, Addis Ababa, Ethiopia; 3 Department of Epidemiology and Biostatistics, Institute of Public Health, University of Gondar, Gondar, Ethiopia; 4 Department of Biomedical Informatics, Vanderbilt University Medical Center, Nashville, USA

**Keywords:** Vaccination, Immunization, Timeliness, Completeness, Ethiopia

## Abstract

**Background:**

Timely vaccination is key to prevent unnecessary childhood mortality from vaccine-preventable diseases. Despite the substantial efforts to improve vaccination completeness, the effort towards timeliness of vaccination is limited with non-attendance and delays to vaccination appointments remaining a big challenge in developing countries. There is also a limited evidence on timeliness of vaccination. Therefore, this study aimed to determine the magnitude and associated factors for timely completion of vaccination among children in Gondar city administration, north-west, Ethiopia.

**Method:**

This study employed a community-based cross sectional study design. A sample size of 821 children aged 12 to 23 months were considered. Two stages random sampling technique was used to select study subjects. To account the effect of clustering, bivariable and multivariable multilevel logistic regression analysis were applied. The measures of association estimates were expressed as adjusted odds ratio (AOR) with their 95% confidence intervals (CIs).

**Results:**

Of the 774 children included for analysis, 498 (64.3%) were fully vaccinated while 247 (31.9%) were fully vaccinated on-time. Caregivers who had secondary education and above (AOR = 2.391; 95% CI: 1.317–4.343), from richest households (AOR = 2.381; 95% CI: 1.502–3.773), children whose mother attended four or more ante natal care visits (AOR = 2.844; 95% CI: 1.310–6.174) and whose mother had two or more post natal care visits (AOR = 2.054; 95%CI:1.377–3.063) were positively associated with on-time full vaccination. In contrary, caregivers aged above 35 years (AOR = 0.469; 95 % CI: 0.253-0.869], being vaccinated at health post (AOR = 0.144; 95%CI: 0.048–0.428) and travelling more than 30 min to the vaccination site (AOR = 0.158; 95%CI: 0.033–0.739) were negatively associated with on-time full vaccination. The random effects indicated that 26% of the variability in on-time full vaccination was attributable to differences between communities.

**Conclusion:**

In this study, untimely vaccination was found to be high. Different individual and contextual factors were found to be associated with on-time full vaccination. Therefore, tailored strategies have to be designed and implemented to address people and the communities where they live. Moreover, timeliness of vaccination should be considered as important indicator of the immunization program performance in Ethiopia.

## Background

Vaccine preventable diseases contribute substantially to under five mortality as well as morbidity [1]. Hence, immunization has become one of the major contributors to public health globally as it prevents communicable disease, particularly in children. Evidence shows that 29% of deaths among under five children are vaccine preventable [2] and being fully vaccinated is associated with 22% lower mortality in children [3].

Immunization averts an estimated 2 to 3 million deaths every year worldwide. However, despite the tremendous efforts made so far, vaccine preventable diseases remain a major health problem among children in developing countries including Ethiopia [4–7].

Completion and timeliness of routine vaccination as recommended by the World Health Organization (WHO) are very crucial for maximum protection of children against specific infections [8]. To effectively control vaccine preventable diseases (VPDs), high immunization coverage is required with a targeted coverage level of 90% recommended by the WHO. In terms of control and prevention of childhood infectious diseases, achieving high vaccination coverage is a necessary; but insufficient indicator to assess the success of immunization program. In order to successfully control and eliminate vaccine-preventable infectious diseases, age appropriate vaccine coverage has to be achieved and maintained [9–12].

However, maintaining a high performance of an immunization program and its quality is challenging, with non-attendance and delays to vaccination appointments remaining a key challenge in developing countries [13, 14]. Worldwide in 2018, about 116.3 million infants (86%) received three doses of diphtheria-tetanus-pertussis (DTP3) vaccine, protecting them against infectious diseases. On the other hand, an estimated 19.4 million infants worldwide were not reached with routine immunization services of which around 60% of these children live in 10 countries, including Ethiopia [15]. As a result, substantial proportions of children in many countries still fail to benefit from all basic vaccines and vaccine preventable diseases still pose a public health risk with the highest rates of child mortality still in Sub-Saharan Africa [16, 17].

Ethiopia launched the immunization program in 1980 and currently provides 11 antigens at birth, 6 weeks, 10 weeks, 14 weeks and 9 months. Despite the fact that immunization services are offered free of charge, full vaccination coverage in Ethiopia is significantly lower than the global target. The 2016 Ethiopian Demographic and Health Survey (EDHS) report showed that only 39% of children received all basic vaccinations [18] and the 2019 mini EDHS report also indicated that the full vaccination coverage has reached 43% with steady increase in vaccination coverage over time [19]. Findings from studies conducted in different settings of Ethiopia showed a wide range coverage of full vaccination from 48.8 to 91.7% [20–26]. On the other hand, the incomplete vaccination coverage ranged from 20.3 to 45.5% [20, 23, 24].

Multiple assessments also reveal that despite relatively high vaccination coverage, there was a gap in the timeliness of children vaccination with substantial delay in age-appropriate vaccination demonstrating that high vaccination coverage does not imply that children are vaccinated according to the schedule [16, 27–36].

Currently, the timing of vaccine administration has received increasing attention in many countries, especially when the level of vaccination coverage is close to that needed for protective herd immunity [10, 29, 37]. Although many studies have measured full vaccination coverage, studies on timely vaccination completion are rare in developing countries particularly in Sub- Saharan Africa (SSA) [16, 31].

To date, timeliness is not routinely used as an indicator to evaluate immunization programs in Ethiopia. Similarly, several studies had examined and documented the vaccination coverage for childhood immunization in Ethiopia with which timeliness had received less consideration [13, 19, 22, 24, 38]. Therefore, this study aimed to determine the coverage and associated factors for timely completion of vaccination among children in Gondar city administration, Northwest, Ethiopia.

## Methods

### Study design, area and period

A community based cross sectional study was conducted from October 22 to November 30, 2018 in Gondar city administration, northwest, Ethiopia. The city administration had an estimated total population of 390, 644 of which 12,149 were under 1 year of age. Gondar city administration has a total of 24 Kebeles (13 urban and 11 rural). In addition, the city administration has a total of 23 public health facilities (one comprehensive specialized hospital, eight health centers and 14 health posts [39].

### Source and study population

The source population were all children aged 12 to 23 months with a history of routine vaccination in Gondar city administration. The study population were those children aged 12 to 23 months with their caregivers living in the eligible households of the selected kebeles and included for this particular study.

### Inclusion and exclusion criteria

Households with at least one live child aged 12 to 23 months and who resided in the study area for at least 6 months prior to the study period were eligible for inclusion in this study. Those children included in this study had a history of routine vaccination. Children having history of vaccination from vaccination campaigns only were excluded.

### Sample size determination 

The required sample size was calculated for both completeness and timeliness of vaccination using single population proportion formula by considering the following assumptions:

- Using proportion of 58.7% for full vaccination [Pilot study], 95% confidence level, 5% margin of error, design effect of 2 and non-response rate of 10%, the sample was 821.
- Using proportion of 37.1% for on time-full vaccination [Pilot study], 95% confidence level, 5% margin of error, design effect of 2 and non-response rate of 10%, the sample was 395.

Hence, we used a sample size of 821 for this particular study.

### Sampling procedure

Two stages sampling technique was used. During the first stage, from the 24 kebeles (13 urban and 11 rural) 40% of the kebeles were considered to be included in this study. From the total kebeles in the city administration, five urban and five rural kebeles were selected proportionally from each stratum using simple random sampling technique.

In the second stage of sampling, at each selected kebele, individual households were selected using systematic random sampling technique. Children in the selected households were further selected. If there were two or more children in the same household, lottery method was used to select one child per household. When there was no eligible child in the selected household, the next household was considered in the study.

### Study variables measurement

#### Dependent variable

On-time full vaccination.

#### Independent variables

Socio-demographic characteristics of the caregiver, health service related characteristics of the mother and contextual factors at community level were considered as independent variables for this study.

Full vaccination was defined as the child vaccination status once an infant has received all recommended vaccines included in the national schedule: a dose of Bacille Calmette Guérin (BCG); three doses of Oral Polio Vaccine (OPV); three doses each of Penta-valent and Pneumococcal Conjugate Vaccine (PCV); one dose of Inactivated Polio Vaccine (IPV); two doses of rotavirus and one dose of measles vaccines by the age of 12 months [5, 22, 26, 40, 41]. On-time vaccination for specific vaccines was defined as vaccine dose administered within 4 days prior [31, 42–45] and within 4 weeks after the recommended age specified in the national immunization schedule [12, 30, 31, 37, 43–49]. On-time full vaccination was also defined as all vaccine doses administered within 4 days prior [31, 42–45] and within 4 weeks after the recommended age specified in the national immunization schedule. Otherwise, it was not considered as on-time full vaccination if at least one vaccine dose was given early, late or missed at all [30, 31, 43, 44, 48, 50–53].

The household socio-economic status was created by principal components analysis (PCA), including variables on asset ownership, housing characteristics and ownership of animals and farming. This was done for rural and urban households separately [54]. Having this, rural and urban households PCA loading scores were merged for household wealth index classification using quintiles. Finally, the merged scores for urban and rural were divided into three quintiles as poor, middle and rich households.

### Data collection tools and procedures

Data collection instrument was adapted from EDHS [18]. Face and content validity has been ensured by a group of six experts. Accordingly, the applicability of the data collection tools and procedures were checked and revised as necessary.

Pilot study was also done out of the study area (in four kebele’s of Bahirdar city administration) before the actual data collection with a sample size of 100. The results of the pilot study were used to determine the minimum sample size for the actual study. The reliability of the data collection instrument was assessed using Cronbach’s alpha (α). From the pilot study the internal consistency estimate for the full vaccination scale was found to be 0.87.

Interviewer-administered data collection instrument was used to collect socio-demographic characteristics, health service related factors and vaccination status of the children. Eight data collectors and two supervisors were recruited for the data collection. Vaccination status and age at vaccination were confirmed by checking the vaccination card kept by caregivers or from the health facility expanded program on immunization (EPI) registers. For children with a vaccination card, the interviewer copied dates of any recorded vaccination on to the data collection instrument. For children without a vaccination card, their vaccination status was verified from the health facility EPI registers.

### Data processing, analysis and parameter estimation methods

#### Descriptive statistics

The data were entered into EPI-data version 3.1 software and transferred to STATA version 14 software for analysis. Prior to the commencement of the analysis data cleaning, labeling, coding and recoding were done for all variables. Frequency and percentages were used to report categorical variables.

### Bivariable and multivariable multilevel regression analysis

At the bivariable multilevel regression analysis, the effect of each individual and community level predictor variables on the outcome variable were checked at significance level of 0.2 [55]. Variables which were significant at the bivariable multilevel logistic regression analysis were considered as candidates for the individual and community level model adjustments. Finally, a significance level of 0.05 was considered for the multivariable multilevel regression models.

### Model specification

This study applied binary logistic multilevel analysis techniques in order to account for the clustering nature of the data and the binary response of the outcome variable. For the bivariable and multivariable multilevel logistic regression analysis the STATA syntax xtmelogit was used. Accordingly, four models containing variables of interest were fitted.

Model-I: was the null model, used to check the variability among the communities without inserting any variable. It’s the first step used to provide evidence whether the data has a justifiable evidence to assess the random effects at the community level. Model-II: was a multivariable model used to adjust individual level variables which were significant at the bivariable multilevel regression analysis. Hence, independent variables which were significant in Model-II were considered as candidates of the final model. Model-III: was also a multivariable model which was used to adjust community level variables which were significant at the bivariable multilevel regression analysis. Community level independent variables which were statistically significant in Model-III were included in the final model. Model-IV: was a multivariable multilevel regression analysis model used to adjust the outcome variable against independent variables which were statistically significant either at Model-II or Model-III. Stepwise model building technique was used for all models.

### Parameter estimation methods

The measures of association (fixed-effects) estimates the association between the likelihood of children to be fully vaccinated on-time and the predictor variables expressed as Adjusted Odds Ratio (AOR) with their 95% Confidence Intervals (CIs). The measures of variation (random-effects) were reported as intra class correlation coefficient (ICC) which is the percentage of variance explained by the community level variables. Proportional Change in Variance (PCV), expresses the change in the community level variance between Model-I (empty model) and the consecutive models (Model-II, III and IV) [56].

### Multicollinearity and interaction effect

The presence of multicollinearity was checked among independent variables using Variance Inflation Factor (VIF) at cut off point of 10 [57]. Similarly, interaction terms between community and individual level variables were tested.

### Comparison of models and model fit statistics

Akakie Information Criterion (AIC) was used to compare the consecutive models. The AIC values for each subsequent models were compared and the model with the lowest value was considered to be the better model [58]**.** Finally, Hosmer-Lemeshow goodness of fit test was used to estimate the goodness of fit of the adjusted final model [55].

### Ethical considerations

Ethical approval was obtained from the University of Gondar Ethical Review Board (IRB) before the commencement of the study. In addition, study permission was sought at all levels of local governmental health administrations. Informed written consent was obtained from each of the caregivers of children for their participation in the study and to access their child vaccination data from health facilities. In the meantime, study participants were informed to withdraw and discontinue participation at any time if they felt discomfort. Moreover, confidentiality assurance was provided to study participants on the information provided by them. Information that was collected for this study was also secured and protected from unauthorized access. At last**,** official permission was requested from the health facilities to access the child vaccination records from EPI registers.

## Results

### Socio-demographic characteristics of caregivers

Out of the 821 children included for this study, the response rate was 98.9%. Thirty eight children with unverifiable records were excluded and a total of 774 children were included for analysis. Almost all (97.8%) of the respondents as a primary caregiver were mothers of the eligible child and 475 (61.4%) were in the age range of 25–34 years. About, 67% of respondents were from urban kebeles. Pertaining to educational status, majority (46%) had secondary education and above while 22% had no education. As indicated in Table 1, 693 (89.5%) of the caregivers were married and 500 (64.6%) were housewife by occupation. With regard to sex of child, 406 (52.4%) of children were males [Table 1].

**Table 1:** Socio-demographic characteristics of caregivers in Gondar city administration, north-west Ethiopia, 2018 [*N* = 774]

Characteristics	Total (%)
**Caregiver age in years**	
≤24	172 (22.2)
25–34	475 (61. 4)
≥35	127 (16.4)
**Marital status**	
Married	693(89.5)
Others	81(10.5)
**Religion**	
Orthodox	707(91.4)
Muslim	53 (6.8)
Others	14 (1.8)
**Residence**	
Rural	252(32.6)
Urban	522(67.4)
**Education**	
No formal education	171(22.1)
Primary	244(31.5)
Secondary and above	359(46.4)
**Sex of child**	
Female	368(47.6)
Male	406 (52.4)
**Occupation**	
Housewife	500 (64.6)
Employed	88 (11.4)
Merchant	115(14.9)
Others	71 (9.1)
**Family size**	
< 5	436 (56.3)
≥5	338(43.7)
**Wealth Index**	
Poor	258 (33.3)
Middle	258 (33.3)
Rich	258 (33.3)

### Health service related characteristics of mothers/caregivers

Majority 469 (60.6%) of caregivers reported that the mother had four or more ante natal care (ANC) visits for the child included for this study. Regarding place of delivery, only 54(6.9%) of deliveries were at home. Sixty eight percent of respondents reported that the mother of the child had two or more post natal care (PNC) visits. Of the included children for the study, 279 (36.1%) were in the first birth order. Four hundred and ninety-two (63.6%) of the caregivers took their child to a health center for vaccination and 391 (50.5%) of respondents reported that the distance to the vaccination site was less than 15 min from their home (Table 2).

**Table 2:** Health service related characteristics of mothers/caregivers in Gondar city administration, north-west Ethiopia, 2018 [*N* = 774]

Variables	Total (%)
**ANC**	
No	61 (7.9)
1–3	244 (31.5)
≥4	469 (60.6)
**Place of delivery**	
Home	54 (6.9)
Health Facility	720 (93.1)
**PNC**	
< 2	243 (31.4)
≥2	531 (68.6)
**Birth order**	
1st	279 (36.1)
2nd-3rd	347 (44.8)
4+	148 (19.1)
**Distance to vaccination site**	
< 15 Minute	391 (50.5)
15-30 Minute	309 (39.9)
> 30 Minute	74 (9.6)
**Place of vaccination**	
Hospital	130 (16.8)
Health post	152 (19.6)
Health center	492 (63.6)

### Over all vaccination status of children

**Fig. 1:**
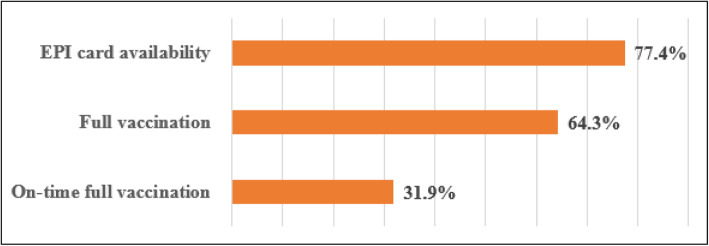
EPI card availability and vaccination status of children in Gondar city, north-west Ethiopia, 2018 [*N* = 774]

Child vaccination card availability during the time of interview was 599 (77.4%) (Fig. 1). Of those caregivers who reported that their child’s vaccination card is available during the time of interview, 551 (91.9%) showed the vaccination cards for the interviewers.

Of the 774 children included for analysis, 498 (64.3%) with 95%CI: (60.9–67.6%) were fully vaccinated while 247 (31.9%) with 95% CI (28.7–35.3%) children were fully vaccinated on-time (Fig. 1).

### Vaccination coverage for specific vaccines

**Fig. 2:**
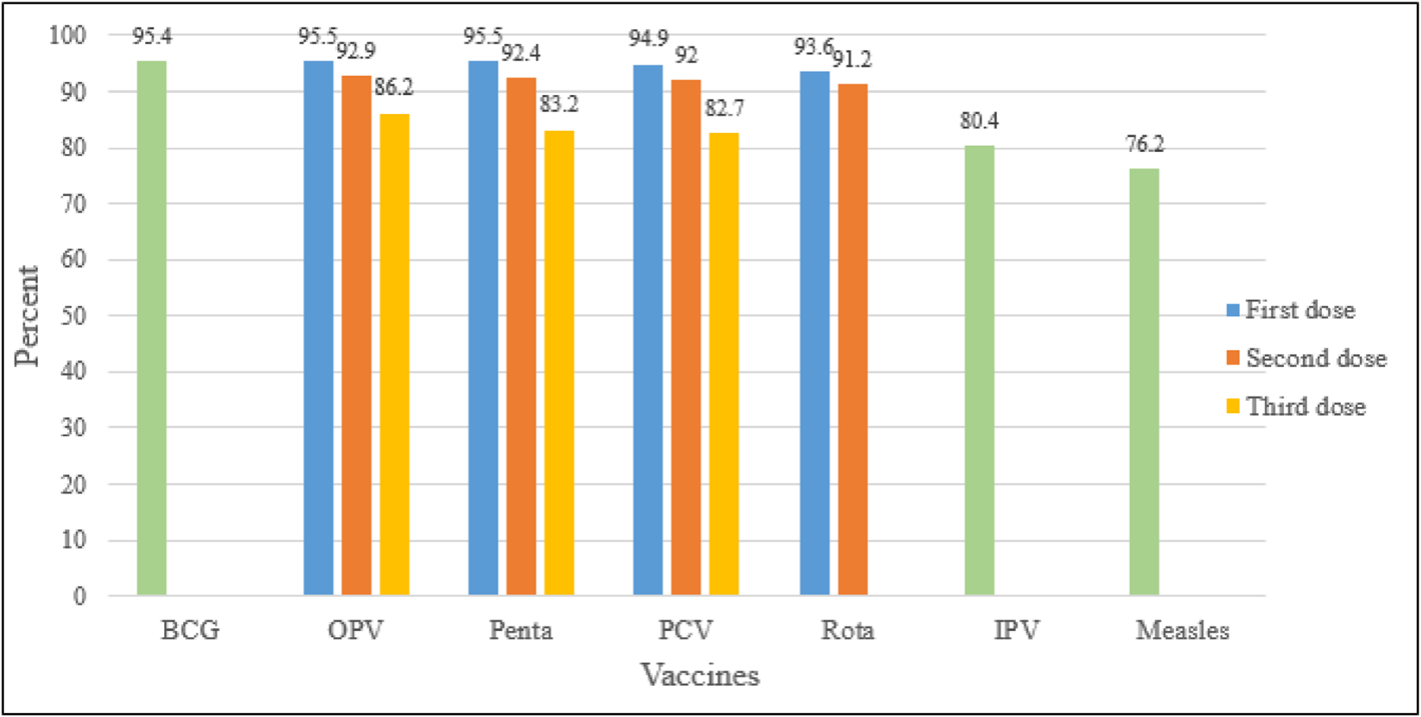
Vaccination coverage for specific vaccines among children in Gondar city, north-west Ethiopia, 2018 [*N* = 774]

Figure 2 below depicted the vaccination coverage for specific vaccines. Coverage for each specific vaccine was calculated from all children included in this particular study. We found that the proportion of children with full vaccinations decreased from Penta I (95.5%) to Penta III (83.2%) and measles (76.2%) vaccine doses subsequently (Fig. 2). The study also indicated that the Pentavalent vaccination drop-out rate was 12.8% and the BCG to measles vaccination dropout rate was 20.1%.

### Timely vaccination for specific vaccines

**Fig. 3:**
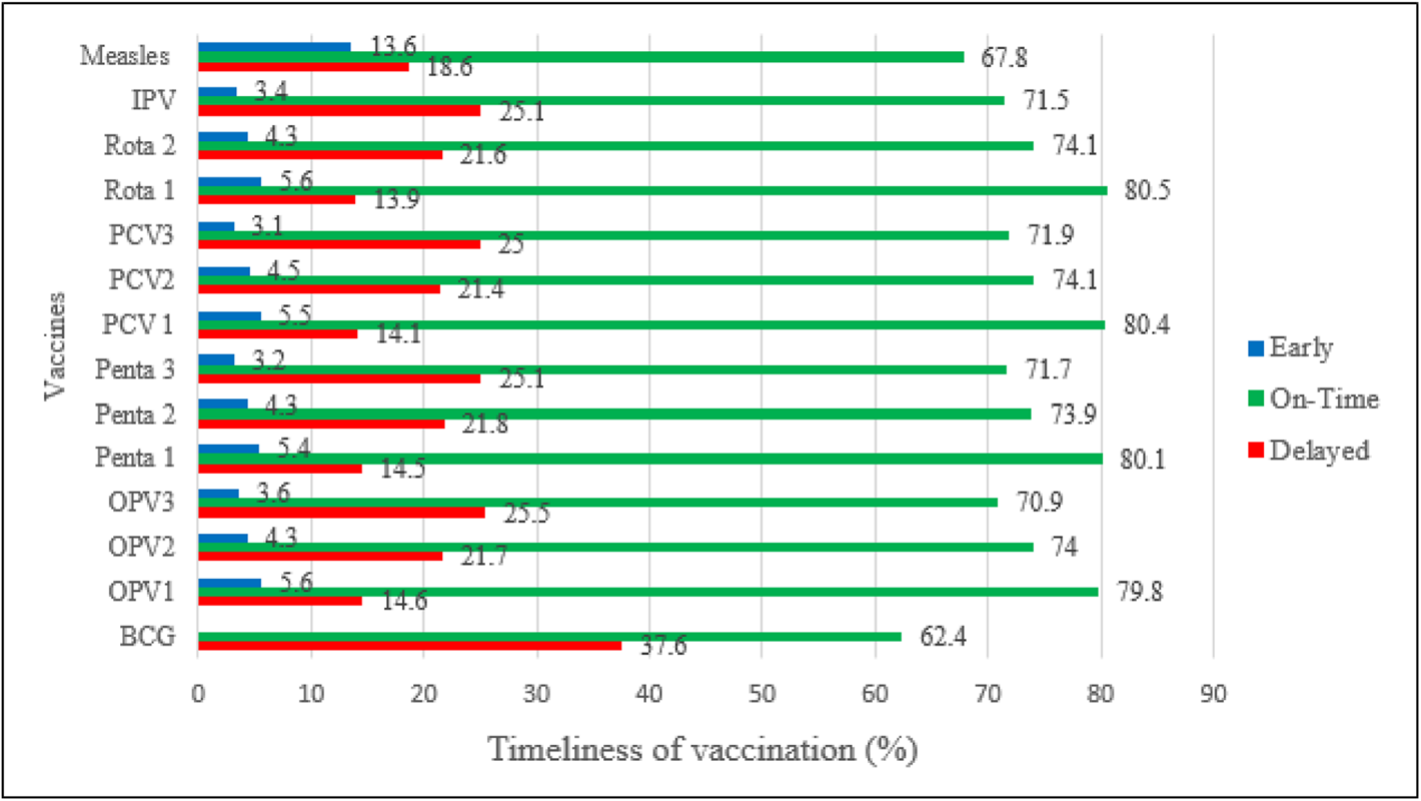
Timeliness of vaccination for specific vaccines among children in Gondar city, north-west Ethiopia, 2018

Timeliness for each specific vaccine was calculated from those children vaccinated for that specific vaccine. As depicted in Fig. 3, timely vaccinations for each vaccine ranged from 62.4% for BCG vaccine to 80.5% for Rota I vaccine. The proportion of children who had received early vaccine doses ranged from 3.1% for PCV3 vaccine to 13.6% for measles vaccine. On the other hand, the proportion of children who had received vaccine doses lately ranged from 13.9% for Rota1 vaccine to 37.6% for BCG vaccines (Fig. 3).

### Attendance to vaccination schedules

Full attendance to vaccination schedules were measured historically by asking the caregivers and objectively measured from vaccination cards and registers. The findings showed that the proportion of full attendance to vaccination schedules measured historically from caregiver’s report was 693(89.5%). On the other hand, the objective measurement from vaccination cards and registers indicated that proportion of full attendance to vaccination visits was 558 (72.1%).

### Reasons for not attending vaccination schedules on-time

**Fig. 4:**
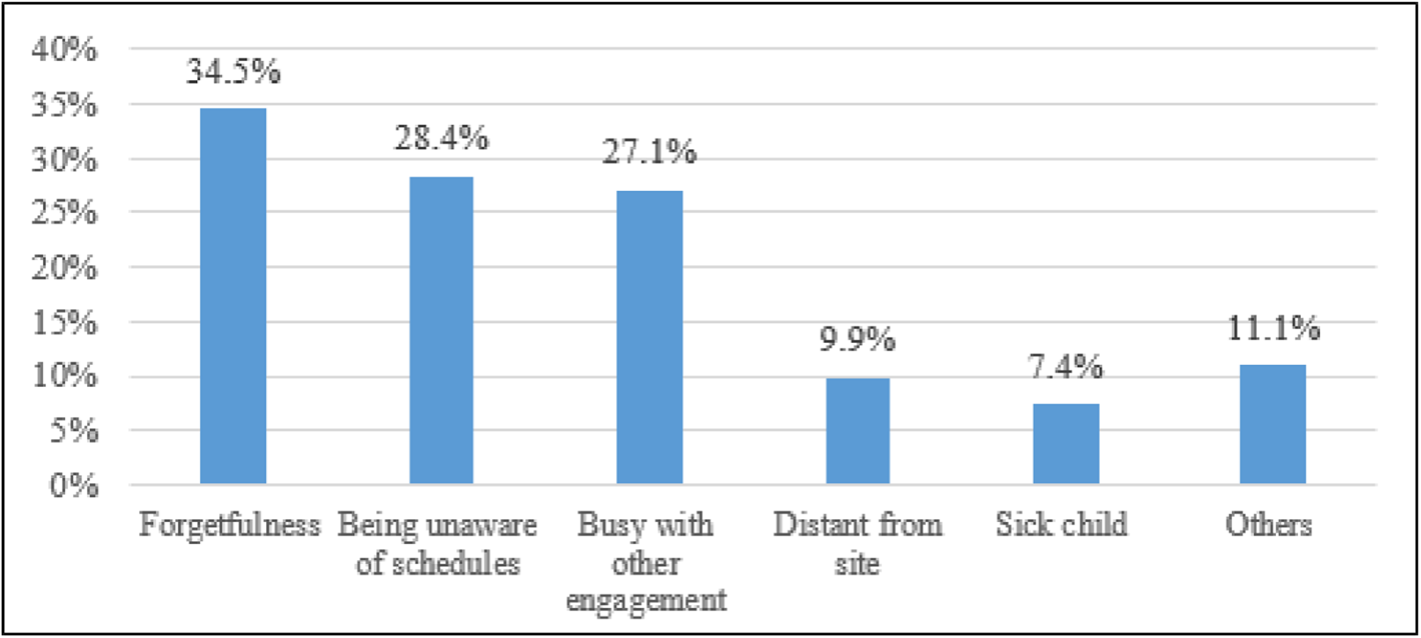
Reasons for not attending vaccination schedules on-time in Gondar city, north-west Ethiopia, 2018 [*N* = 81]

The reasons for not attending vaccination schedules were mentioned by those 81 caregivers who reported that their attendance to the vaccination schedules were not complete as scheduled. Among the reasons for not attending vaccination schedules on-time, 34.5% were due to forgetfulness, 28.4% being unaware of the schedules and 27% being busy with other engagements to show up in vaccination schedules (Fig. 4).

### Multilevel logistic regression analysis

The fixed and random effects for on-time full vaccination are presented in Table 3. The ICC in the empty model implied that 25.7% of the total variance in on-time full vaccination was attributed to differences between communities (Table 3).

**Table 3:** Multilevel regression analysis of factors associated with on-time full vaccination in Gondar city, north-west Ethiopia

Fixed effects of individual and community level variables		Model-I	Model-II AOR [95%CI]	Model-III AOR [95%CI]	Model-IV AOR [95%CI]
**Age of caregivers**	≤24 years		1	–	1
	25–34 years		1.012 [0.669,1.531]	–	1.031[0.681,1.561]
	≥35 years		0.441[0.238,0.813]	–	0.469[0.253,0.869]
**Education of caregivers**	No education		1	–	1
	Primary		1.901[1.012,3.571]	–	1.786[0.954,3.343]
	Secondary and above		2.587[1.407,4.756]	–	2.391[1.317,4.343]
**Wealth Index**	Poor		1	–	1
	Middle		1.419[0.915,2.201]	–	1.494[0.976,2.287]
	Rich		2.269[1.419,3.627]	–	2.381[1.502,3.773]
**ANC**	No		1	–	1
	1–3		1.416[0.627,3.201]	–	1.404[0.618,3.191]
	4+		3.139[1.456,6.764]	–	2.844[1.310,6.174]
**PNC**	< 2		1	–	1
	≥2		2.067[1.373,3.109]	–	2.054[1.377,3.063]
**Place of vaccination**	Hospital		–	1	1
	HP		–	0.103[0.036,0.289]	0.144[0.048,0.428]
	HC		–	0.809[0.511,1.282]	1.011[0.612,1.671]
**Distance to vaccination site (minute)**	< 15 min		–	1	1
	15–30 min		–	0.661[0.469,0.933]	0.746[0.516,1.080]
	> 30 min		–	0.155[0.034,0.701]	0.158 [0.033,0.739]
**Random effects**					
**Random effect**		**Model-I**	**Model-II**	**Model-III**	**Model-IV**
Community variance (SE)		1.14 (0.62)	1.01 (0.59)	0.069 (0.08)	0.079 (0.093)
ICC (%)		25.7%	23.5%	2.1%	2.4%
PCV (%)		Ref	11.4%	93.9%	93.1%
**Model comparison statistics**					
**Model comparison**		**Model-I**	**Model-II**	**Model-III**	**Model-IV**
**Log likelihood**		− 447.77844	−407.19174	− 432.57621	− 393.93011
**AIC**		899.5569	836.3835	877.1524	817.8602

In Model-II only individual level variables were added. In this model the variables age of caregiver, marital status, religion, occupation, family size, sex of child, caregiver education, birth order, ANC, place of delivery, PNC and wealth index were included. With this, marital status, religion, family size and sex of child were insignificant at the bivariable regression analysis. Finally, the variables occupation, birth order and place of delivery were statistically insignificant at Model-II. The results showed that caregiver’s age, caregiver’s education level, household wealth index, antenatal care visits and post natal care visits were significantly associated with on-time full vaccination in Model-II. The ICC in Model-II indicated that, 23.5% of the variation in on-time full vaccination was attributable to differences across communities. As shown by the PCV, 11.4% of the variance in on-time vaccination across communities was explained by the individual level characteristics (Table 3).

In Model-III only community level variables were added. In model-III the community level characteristics residence, distance to vaccination site and place of vaccination were included. At the bivariable regression analysis all the three variables were statistically significant. In model-III, the variable place of residence became statistically insignificant. The results in Model-III revealed that place of vaccination and distance to the vaccination site were significantly associated with on-time full vaccination. The ICC in Model-III implied that differences between communities account for about 2.1% of the variation in on-time full vaccination. In addition, the PCV indicated that 93.9% of the variation in on-time full vaccination between communities was explained by community level characteristics (Table 3).

Model-IV, the final model included both the individual and community level characteristics simultaneously. After controlling for other individual and community level factors, caregivers aged above 35 years were 53% less likely (AOR = 0.469; 95% CI: 0.253–0.869] to complete their child vaccination on-time as compared to those caregivers aged 25 years and less. The study also indicated that caregivers who had secondary education and above were 2.4 times (AOR = 2.391; 95% CI: 1.317–4.343) more likely to complete their child vaccination on-time as compared to those who had no education after controlling for other variables. After holding other factors constant, caregivers from richest households had 2.4 times higher chance of completing their child vaccination on-time (AOR = 2.381; 95% CI: 1.502–3.773) as compared to caregivers from poorest households (Table 3).

Looking at ANC, children whose mothers had attended four and above ante natal care visits were 2.8 times (AOR = 2.844; 95% CI: 1.310–6.174) more likely to complete their child vaccination on-time as compared to those children whose mothers had no antenatal care checkups. Keeping other variables constant, children whose mothers had two and more PNC visits were 2 times more likely (AOR =2.054; 95%CI:1.377–3.063) to fully vaccinate their child on-time as compared to their counterparts (Table 3).

Pertaining place of vaccination, those caregivers who vaccinated their child at health posts were 86% (AOR = 0.144; 95%CI: 0.048–0.428) less likely to fully vaccinate their child on-time as compared to those who vaccinated their child in hospital. In terms of distance to vaccination site, those caregivers who travelled more than 30 min to the vaccination site were 84% (AOR = 0.158; 95%CI: 0.033–0.739) less likely to fully vaccinate their child on-time as compared to those who travelled less than 15 min to the vaccination site (Table 3).

As shown by the estimated ICC in model-IV, 2.4% of the variability in on-time full vaccination was attributable to differences between communities. The PCV indicated that, 93.1% of the variation in on-time full vaccination across communities was explained by both individual and community level factors included in model-IV (Table 3).

### Multicollinearity and interaction effect

Multicollinearity was checked for those variables included in the final model using VIF. Accordingly, the VIF for all predictor variables included in the final model was below 10 indicating absence of multicollinearity among the predictor variables**.** Similarly, interactions between community and individual level variables were tested and there was no statistically significant cross-level interaction.

### Comparison of models and model fit statistics

Akakie Information Criterion (AIC) was used to compare the models. The AIC values for each subsequent models were compared and Model-IV with lowest value of AIC was considered to be the better model (Table 3).

Finally, goodness of fit test was done for the final model. The Hosmer and Lemeshow test was statistically insignificant indicating that the final model fits the data very well (*P*-value: 0.279).

## Discussion

Timely vaccination is very important to get the maximum benefit of the vaccine. As the health system is mostly focusing on the completion of vaccines, timely completion of recommended vaccines is important for evaluating the effectiveness of immunization programs in Ethiopia. In this study a total of 774 children nested within 10 clusters were included in the analysis after excluding 38 unverifiable records. The analysis showed that timely vaccination coverage is low in the study area. The results of the study also indicated that individual and community level factors were significantly associated with on-time full vaccination.

The findings of this study indicated that around two-third of children were fully vaccinated which is far behind the national target [5]. This finding is also relatively lower than the findings of studies in Dessie [21], Woldia [22] and Markos [26] towns. This could be explained by the difference in information source for outcome measurement where we have used objective measures to ascertain vaccination status of children from the EPI cards and health facility registers only. In this study, one third of children had not completed their vaccination and the coverage rate for specific vaccines declined for subsequent doses of vaccines with high dropout rates. This finding corroborates with other evidences where missing measles and third doses of polio and pentavalent vaccine were the main reason for not being fully vaccinated [3, 24, 43]. In addition, the study indicated different coverage’s for specific vaccines provided in the same vaccination visits. This finding is in line with other studies [21, 26]. This might be related with unavailability of some specific vaccines or supplies especially for the newly introduced vaccines like Rota and PCV during vaccination sessions. Service availability and readiness assessment report of 2018 also showed that availability of vaccines in health facilities ranges between 28 and 30% [59].

In terms of timeliness, we found that that almost two-third of the children were not vaccinated on-time either being early or late from the recommended time schedules. These findings corroborate the findings of other studies most of which reported higher proportion of delays in child vaccinations [10, 13, 27, 33, 49, 60–64] This study also pointed out that only half of fully vaccinated children were timely for all the vaccine doses. This indicates that merely relying on vaccination coverage overestimates population immunity, as it does not account for delays in protection and extended susceptibility to preventable diseases. Another reports in the literature also concur that timely vaccination coverage is a better performance metric for routine immunization services than crude vaccination rates alone [53, 61]. As such, untimely vaccinations are likely to contribute greatly to the preventable disease burden in Ethiopia, allowing for transmission among those in the age group at which they are the most vulnerable to severe disease.

In the present study timely vaccination coverage was not only low, but also it declines as one goes from penta I to penta III and measles vaccine doses which is consistent with other evidences [32, 45, 46]. This might be due to increased caregiver’s workload with other activities while the child gets older and thereby might not remember vaccination appointments of their children.

This study also found that a substantial number of children started their routine vaccination much earlier than the recommended age. Similar result has been reported elsewhere [43]. We found a high proportion of children vaccinated before 9 months of age for measles which may be related with the measles open vial policy issues in which, the measles multi-dose vial has not been opened daily unless sufficient numbers of children were found in the vaccination site; be it in a static or outreach setting. According to the Advisory Committee on Immunization Practice recommendation, these earlier vaccine doses leads to low sero-conversion rates with less child protection from vaccine-preventable disease and the vaccine dose should be repeated [42].

This study also demonstrated that there was a delay in all vaccine types showing that many Ethiopian children are receiving their vaccinations later than recommended, leaving them unnecessarily vulnerable to disease for extended periods. The implication of delay in receipt of vaccines is that a pool of children with incomplete or no immunization may build up [62]. The presence of such a pool of susceptible children predisposes to outbreaks of vaccine preventable diseases [13].

This study finally assessed the individual and community level factors associated with on-time full vaccination. At the individual level the variables age of caregiver, educational status, household wealth index, ANC and PNC service utilization were significantly associated with on-time full vaccination. The study showed that, as the caregivers get older the odds of timely full vaccination decreases. This finding is consistent with findings from Belgium [10], China [30] and Saudi Arabia [37]. This difference could be explained by the reason that the younger caregiver would possibly have a better utilization of health care which may lead to an increased probability of vaccinating their child on-time [30]. On the other hand no significant effect was reported from studies in Burkina Faso [31] Uganda [13] and Pakistan [52].

On-time full vaccination coverage was higher among children of caregivers with high educational level. Several studies also support the finding of higher educational level being related to timely adherence to the vaccination schedules [13, 16, 31, 32, 50, 51, 63, 65]. The possible reason for this may be related with the fact that the low education level can hinder the caregiver’s communication with health workers and might influence caregiver’s awareness to seek and take advantage of public health services including child vaccination.

Household wealth index was also found to be a significant predictor of on-time full vaccination in this study, with a better timeliness of vaccination among children from the richest households. This finding is consistent with findings from other countries [10, 13, 16, 50, 51, 61, 66]. Though immunization services in Ethiopia are completely free of charge, the indirect cost of vaccination, such as income loss and transportation cost, might be associated with the low demand for vaccination especially for poorest households [50].

ANC visit of four or more at the health facility predicts better timely completion of child vaccination as it has been reported from other study settings [13, 61, 65]. It is possible that mothers who attended ANC visits at health facilities may be more frequent users of health facilities and services including vaccination for children. In this study on-time full vaccination coverage was high among children whose mothers attended postnatal care [43]. This is expected as they can get counselling about vaccination during PNC visits and their children have more chances of getting the vaccines than those who do not make any follow-up contact for PNC service.

At community level, the variables distance to the vaccination site and place of vaccination were significantly associated while place of residence didn’t show significant association with on-time full vaccination. In this study, distance to the vaccination site was negatively associated with on-time full vaccination. This finding is consistent with studies in Burkina Faso [31], China [30] and Tanzania [36]. Thus, this further strengthened the argument that the time spent to reach the vaccination site expenses a high opportunity cost to caregivers by creating the need for multiple visits, especially when vaccine vials were not opened for a small number of children like BCG and measles vaccines.

The findings of this study also indicated that timely vaccination was more likely if the child was vaccinated in hospitals than health posts. This study is consistent with a study in Lebanon [60]. This may reflect that health services, mainly having a better utilization of vaccination services, were more preferable and accessible to caregivers. National reports also indicated that child vaccination as an outreach service was not commonly offered on daily basis which is the case at health post level [59, 67].

In our study, place of residence have no significant association with on-time full vaccination. In Ethiopia, urban areas might have easier access to health services and better transportation available whereas the introduction of the health extension program might have increased access to vaccinations in rural areas with well-established rural outreach immunization efforts, which may account for the insignificant results in this study. This finding corroborates with a finding from Pakistan where residence has no effect on timeliness of vaccination [52]. This finding is inconsistent with other findings from Bangladesh [50], Vietnam [51] and Nigeria [68] where children from urban areas have better timeliness for vaccination. This was justified by the reason that health facilities are more proximal to clients in the urban community than the rural community. On contrary, in a study from Burkina Faso children from rural have better timeliness for Penta III and measles vaccination [31]. It was explained that although the urban area has a better health infrastructure compared to the surrounding villages, caregivers need to take their children to the health facilities by their own initiative, while rural villages are visited by an outreach vaccination team each month.

In addition, we found evidence of clustering effects of timely full vaccination at community (Kebele) level, such that children from the same communities tended to have similar vaccination status. This suggests that public health programs designed to improve timeliness of vaccination should address people and the communities in which they live [69].

The results of this study should be interpreted taking in to account the following limitations. Though Gondar city administration has urban and rural kebeles, the study was restricted to children having a history of routine vaccination in one city administration. So, the study findings might not be generalizable to children in all regions across Ethiopia. Our study participants might have also introduced recall bias in remembering the frequency of maternal health service utilization factors and the reasons for untimely vaccinations. To reduce recall bias, we ascertained vaccination outcomes objectively from EPI cards and EPI registers of health facilities. Despite these limitations, this study is a community-based survey that would be more representative of the children population. Similarly, the analysis considered the clustered nature of the data by applying multi-level modeling.

### Implications for practice and research

The evidence presented in this study highlighted that strengthening immunization program requires special efforts directed towards the inclusion of timeliness of vaccination as another indicator to monitor the performance of the EPI program in Ethiopia. Future strategies and studies should also develop and test intervention programs to improve timely vaccination for children in developing countries like Ethiopia. In addition, conducting a nationally representative survey about timeliness of vaccination is important to monitor the quality of the EPI program.

## Conclusion

In this study, full vaccination coverage was relatively low and untimely vaccination was found to be high. At individual level the variables caregiver’s age, caregiver’s education, household wealth index, ANC and PNC service utilization were the significant factors affecting on-time full vaccination. In addition, at community level the variables distance to vaccination site and place of vaccination were the significant predictors of on-time full vaccination. Therefore, health facility vaccinators should focus on timeliness of vaccination to minimize early and delayed administration of vaccines. Moreover, targeted interventions should be implemented among the older, uneducated and caregivers with low socio-economic status. In addition, strengthening the quality of ANC and PNC services is also important. At health post level, emphasis should be given for daily vaccination service provision.

## Data Availability

The datasets used for this particular study will be available from the corresponding author up on reasonable request.

## Abbreviations

AIC: Akakie Information Criterion
ANC: Ante Natal Care
AOR: Adjusted Odds Ratio
BCG: Bacille Calmette Guérin
CI: Confidence Interval
DTP: Ddiphtheria-Tetanus-Pertussis
EDHS: Ethiopian Demographic and Health Survey
EPHI: Ethiopian Public Health Institue;
EPI: Expanded Program on Immunization
FMOH: Federal Ministry of Health
ICC: Intra Class Correlation Coefficient
IPV: Inactivated Polio Vaccine
OPV: Oral Polio Vaccine
PCA: Principal Component Analysis
PCV: Proportional Change in Variance
PNC: Post Natal care
VIF: Variance Inflation Factor
VPDs: Vaccine Preventable Diseases
WHO: World Health Organization
